# Catheter-guided anvil insertion for circular stapler esophagojejunal anastomosis: a novel technique in laparoscopic total gastrectomy

**DOI:** 10.1007/s13304-024-01753-2

**Published:** 2024-03-07

**Authors:** Zeyao Ye, Pengfei Yu, Yang Cao, Yian Du

**Affiliations:** Department of Gastric Surgery, Zhejiang Cancer Hospital, Hangzhou Institute of Medicine (HIM), Chinese Academy of Sciences, Hangzhou, 310022 Zhejiang China

**Keywords:** Catheter-guided stapler anvil insertion, Laparoscopic total gastrectomy, Esophagojejunal anastomosis, Circular stapler, Feasibility, Safety, Short-term outcomes

## Abstract

**Objective:**

This study evaluates feasibility, safety, and short-term outcomes of employing the catheter-guided stapler anvil insertion technique for esophagojejunal anastomosis using a circular stapler during laparoscopic total gastrectomy (LTG).

**Materials and methods:**

From September 2021 to April 2023, the catheter-guided stapler anvil insertion technique was employed in 80 patients undergoing laparoscopic total gastrectomy (LTG) for esophagojejunal anastomosis. A modified D2 dissection, according to the en bloc technique, was performed in the patients. Subsequently, a longitudinal incision, approximately 2 cm in length, was made on the anterior wall of the esophagus, about 2 cm above the tumor. The transection line was pre-marked with blue dye along the esophagus's minor axis, and the tail of the anvil was capped with a 10-cm length of catheter (F14 d4.7 mm). The surgeon secures the head of anvil and carefully inserts it into the esophagus, ensuring that only a 5-cm segment of the catheter remains outside the esophagus. A linear cutter was employed to transect and seal the lower end of the esophagus. Subsequently, esophagojejunostomy was performed under laparoscopic guidance using a circular stapler.

**Results:**

Among patients undergoing esophagojejunal anastomosis with the new technique, postoperative complications included pneumonia or pleural effusion in 14 patients (17.5%), anastomotic stenosis in 3 patients (3.75%), abdominal infection in 2 patients (2.5%), and intestinal obstruction in 1 patient (1.25%). No instances of anastomotic leakage, anastomotic bleeding, or deaths were recorded. All patients experiencing complications improved with conservative treatment, without the need for secondary surgery.

**Conclusion:**

The catheter-guided stapler anvil insertion technique is demonstrated to be a safe and effective method for esophagojejunostomy, potentially reducing the occurrence of anastomotic leakage.

## Introduction

Gastric cancer, a common malignant neoplasm of the gastrointestinal tract, ranks fifth in global incidence and third in mortality among malignancies [1]. Although the global incidence of gastric cancer is declining, the incidence of proximal gastric cancer shows a consistent upward trend [2]. Laparoscopic gastrectomy is widely accepted for gastric cancer surgery, especially in early-stage tumors. Advancements in minimally invasive surgery have led to the standardization of surgical approaches and lymph node dissection techniques.

A wealth of studies demonstrate that laparoscopic total gastrectomy (LTG) achieves oncological outcomes comparable to open total gastrectomy [3]. However, constructing an effective esophagojejunostomy is a central and challenging aspect of LTG, especially for cases with tumors in the proximal stomach or esophageal involvement [4]. Esophagojejunostomy, a crucial step in reconstructing the gastrointestinal tract after total gastrectomy, exerts a significant influence on postoperative outcomes.

Traditionally, esophagojejunostomy following total gastrectomy (TG) is performed using the Roux-en-Y anastomosis technique [4]. In laparoscopic procedures, both linear staplers and circular staplers have been employed for esophagojejunostomy [5]. Linear staplers facilitate intracorporeal trocar insertion, eliminate the need for purse-string sutures, ease instrument manipulation, and reduce anastomotic stenosis risk. However, linear staplers require preserving a longer segment of the distal esophagus for anastomosis, limiting the surgical margin. Additionally, the overlapping of the esophagus and jejunum in linear anastomosis creates significant tension at the anastomotic site, especially at the stapler's apex.

Conversely, circular staplers provide enhanced tension reduction and a better esophageal margin. However, the use of circular staplers has limitations; they cannot be introduced through a trocar and require an additional minilaparotomy for intracorporeal placement, which compromises the advantages of laparoscopic surgery. Additionally, applying excessive force while inserting the circular stapler anvil can cause mucosal lacerations in a non-compliant esophagus, potentially leading to severe anastomotic leakage [6]. Furthermore, constrained workspace and limited instrument maneuverability during intracorporeal introduction may cause the circular stapler to dislodge from the jejunum, leading to secondary injury.

Multiple techniques have been developed to overcome challenges in circular stapler anvil insertion, including the purse-string technique, transoral insertion using the OrVil™ device, and the reverse puncture technique. However, each method has its limitations, such as technical complexity, higher risks, or increased costs.

In this study, we introduce a modified technique that utilizes a catheter (F14 d4.7 mm) for guidance, combined with a novel approach for circular stapler anvil insertion, to facilitate successful esophagojejunostomy in LTG. The study aims to assess the feasibility, safety, and short-term outcomes of the transabdominal stapler anvil insertion technique, offering a potential solution for overcoming challenges in esophagojejunostomy during LTG.

## Materials and methods

### General information

The study analyzed data from 80 patients who underwent laparoscopic radical total gastrectomy with esophagojejunostomy at the Zhejiang Cancer Hospital from September 2021 to April 2023. The inclusion criteria included: (1) gastric adenocarcinoma confirmed by preoperative gastroscopy and histopathological biopsy; (2) tumors located in the esophagogastric junction or gastric body, classified as Siewert II or III if located at the junction; (3) clinical tumor stage of IIIa or lower according to the AJCC 8th edition staging system; (4) postoperative pathological examination confirming gastric adenocarcinoma; and (5) scheduled total gastrectomy. The exclusion criteria were: (1) distant metastasis confirmed by preoperative examinations; (2) intraoperative confirmation of distant metastasis or presence of severe malnutrition, cardiopulmonary diseases, or coagulation disorders; and 3) severe internal medical conditions such as cardiopulmonary diseases, which make the patient unable to tolerate anesthesia and surgery, revealed by preoperative examinations. All procedures were performed laparoscopically without the need for conversion to open surgery.

### Methods

**Fig. 1:**
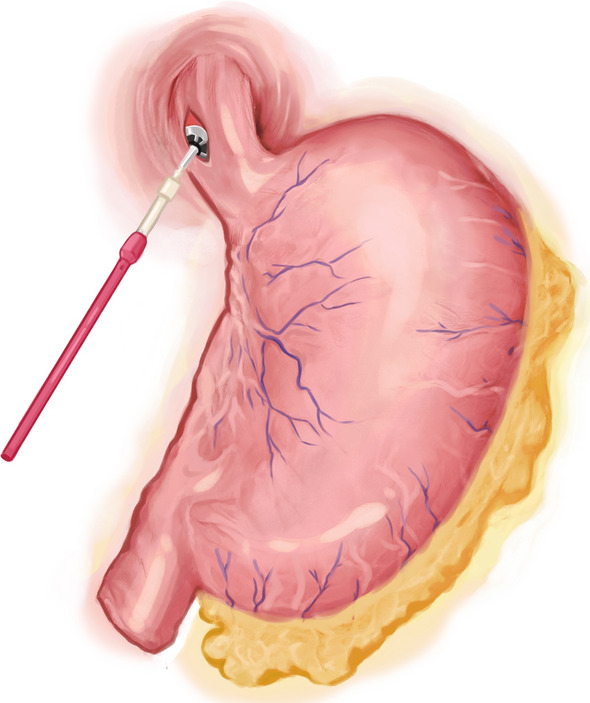
To insert the circular stapler anvil

**Fig. 2:**
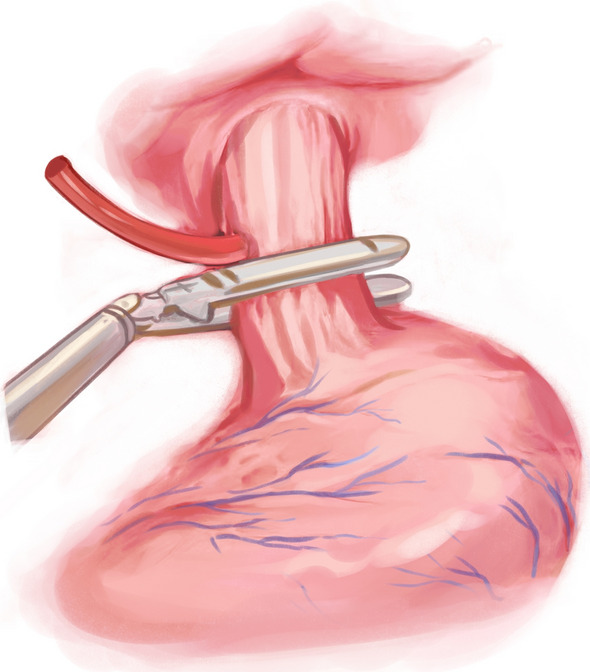
To transect the esophagus at the stapler entry point

**Fig. 3:**
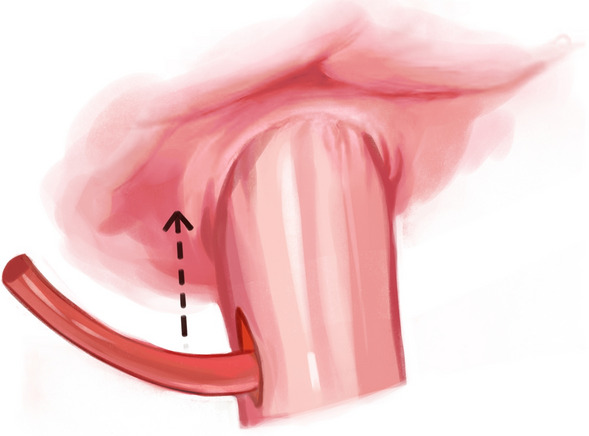
Perform an additional upward incision

**Fig. 4:**
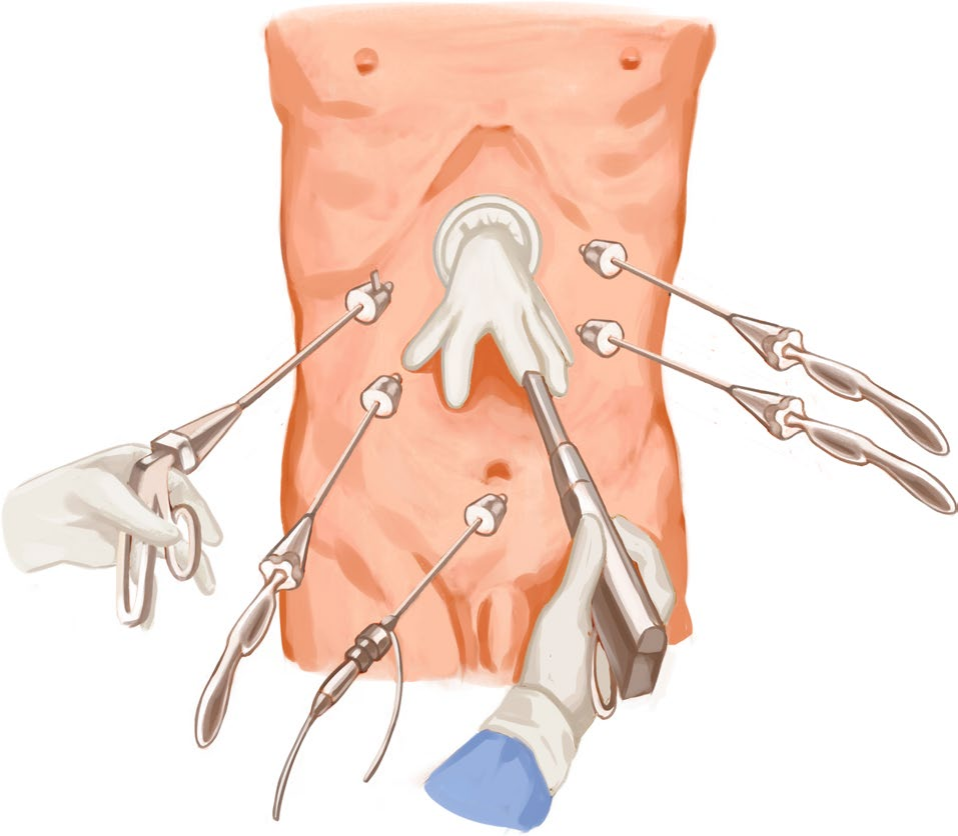
Restore pneumoperitoneum

After performing a modified D2 dissection, the lower segment of the esophagus was fully exposed. A 5-cm margin was left above the resection line, and this line was marked with blue dye along the minor axis of the esophagus. A catheter (F14 d4.7 mm) was attached to the tail of the circular stapler anvil (Fig. 1). The catheter was pre-tractioned and inserted to ensure it was adequately secure. We utilized a linear cutter (ETHICON Echelon Flex 45 mm) to transect the esophagus at the stapler entry point (Fig. 2). Subsequently, we extracted the specimen for margin assessment. In case of insufficient margins after specimen removal, we performed an additional upward incision in the right wall of the esophagus. A catheter aided in elevating the anvil within the esophagus for subsequent resection (Fig. 3). Following that, with assistance through the minor incision, we performed an end-to-side jejunojejunostomy. To restore pneumoperitoneum, a sterile glove was placed over a wound protector. The primary body of the circular stapler (COVIDIEN Premium Plus CEEA 25 mm) was introduced into the jejunum using a sectioned finger cot and secured in place by tying (Fig. 4). With laparoscopic guidance, we carefully maneuvered the catheter to guide the anvil head along the esophageal pathway. Pre-lubrication facilitated this process. The assistant secured the anvil's head with atraumatic forceps, while the lead surgeon detached the catheter from the anvil by pulling. After restoring pneumoperitoneum, the circular stapler was reassembled. After removing gloves and the stapler, pneumoperitoneum was re-established. The surgery concluded with laparoscopic sealing of the remaining opening of the intestinal tube, completing the reconstruction of the digestive tract.

### Evaluation indicators

The following parameters were recorded: the duration of the operation, the time taken to complete the anastomosis, the amount of blood loss during the surgery, TNM staging, the distance of the margins, the time post-operation for the patient to pass gas, the time post-operation before the patient could resume oral intake, and the length of the postoperative hospital stay. Additionally, the incidence of complications, such as leakage, bleeding, or stenosis, at the anastomosis site, as well as pleural effusion and pneumonia, was documented.

### Statistical analysis

Data were subjected to analysis employing SPSS version 25.0. Continuous variables were expressed as mean ± standard deviation (x ± s), while categorical variables were represented in percentages.

## Results

Table 1 contains basic demographic information and clinical histories of the patients, such as age, gender, body mass index, and preoperative health conditions. Table 2 details the surgical data and postoperative outcomes. The mean duration of the operation was 234.16 ± 25.26 min, with an anastomosis completion time of 31.20 ± 8.62 min and an average intraoperative blood loss of 53.75 ± 19.12 ml. There was no need to convert to open surgery for any patient. Postoperative complications included pneumonia or pleural effusion in 14 patients (17.5%), anastomotic stenosis in 3 patients (3.75%), abdominal infection in 2 patients (2.5%), and intestinal obstruction in 1 patient (1.25%). No instances of anastomotic leakage, anastomotic bleeding, or deaths were recorded. On average, patients resumed their diet after 5.20 ± 0.56 days, passed gas after 2.64 ± 0.60 days, and were hospitalized for 8.18 ± 2.24 days post-surgery.

**Table 1:** Basic demographic information and clinical histories

Variable	Number/Value
Gender	
Male	51
Female	29
Age(years)	64.96±10.26
BMI	22.82±2.91
Preoperative Comorbidities	
Hypertension	21
Diabetes	10
Coronary Heart Disease	2

**Table 2:** Surgical data and postoperative outcomes

Variable	Number/Value
Operative Time (min)	234.16±25.26
Anastomosis Time (min)	31.20±8.62
Intraoperative Blood Loss (ml)	53.75±19.12
Tumor Location	
Gastroesophageal Junction Tumors Siewert Type II	14
Gastroesophageal Junction Tumors Siewert Type III	20
Gastric Body and Gastric Fundus Tumors	46
Resection Margins	
<2cm	0
2~5cm	51
>5cm	29
T Stage	
1a	8
1b	15
2	30
3	15
4a	12
N Stage	
0	27
1	22
2	24
3	7
Mean Number of Lymph Nodes Dissected	35.98±13.47
Mean Duration until Resumption of Diet	5.20±0.56
Mean Duration to Pass Flatus	2.64±0.60
Mean Duration of Postoperative Hospitalization	8.18±2.24
Postoperative Complications	
Pneumonia/Pleural Effusion	14(17.5%)
Anastomotic Stenosis	3 (3.75%)
Abdominal infection	2 (2.5%)
Intestinal Obstruction	1 (1.25%)
Anastomotic fistula	0 (0%)

## Discussion

Laparoscopic surgery has witnessed substantial advancements in both skills and equipment over the past few decades. Laparoscopic distal gastrectomy (LDG) has become a widely used procedure for the early treatment of gastric cancer, owing to its evident benefits. However, with the increasing incidence of advanced gastric cancer, total gastrectomy (TG) is often required. At present, there is no standardized method for reconstructing the digestive tract in laparoscopic TG, and the existing techniques have certain drawbacks. Therefore, developing a straightforward and safe reconstruction method is imperative for further advancement in laparoscopic TG. The innovation of stapling devices has undoubtedly played a significant role in the evolution of laparoscopic gastrointestinal surgery, particularly in LTG. Two principal types of mechanical anastomosis are utilized in esophagojejunostomy during LTG: circular stapler anastomosis and linear stapler anastomosis [5].

A number of studies have made comparisons between linear and circular stapling techniques. A recent network meta-analysis indicates comparable risks of postoperative anastomotic leak and stenosis when comparing overlap, functional end-to-end anastomosis, single staple technique, hemi-double staple technique, and OrVil® techniques [7]. Nevertheless, the study was unable to assess the volume and experience of the operating surgeons. One such study by Sejin Lee and colleagues revealed that while the anastomotic leakage rates were comparable for both techniques, linear stapling was associated with a lower incidence of anastomotic stenosis compared to circular stapling [8]. Nonetheless, these studies come with certain limitations. For instance, in such retrospective studies, there is often a tendency to opt for linear stapler anastomosis when the residual esophageal stump is sufficiently long (4.5–6 cm), while circular stapling is typically used in cases where the residual stump length is inadequate, which introduces a bias. The predominant techniques employed in linear stapling include the overlap method and the "Π" anastomosis method. The linear stapling technique is characterized by several advantages, such as operational simplicity, reduced anastomosis duration, lower risk of anastomotic stenosis, and enhanced visualization, during laparoscopy. Furthermore, the technique is not constrained by the diameter of the esophagojejunal lumen. Nonetheless, there are drawbacks to using linear stapling in LTG. One limitation is that it necessitates a longer esophageal stump, which in turn restricts the incisal margin. Additionally, when the anastomosis plane is situated above the esophageal hiatus, the surgery is conducted within a confined thoracic cavity, which restricts the visual field. Moreover, the manipulation involving pulling and folding of the jejunal limb can result in elevated tension at the site of anastomosis. To mitigate the tension, it is often necessary to perform mesentery trimming, which additionally requires careful consideration of the course of the jejunal vascular arcade. However, the effectiveness of mesentery trimming performed laparoscopically can be constrained by suboptimal lighting and restrictive angles, making the procedure more challenging and risk-prone, particularly in patients with obesity or cases that present variations in mesenteric vasculature. Unlike the uniform tension distribution achieved through circular stapling, linear stapling involves overlapping a segment of the jejunum with the distal esophagus. This results in an uneven distribution of tension across the anastomosis site, particularly at the staple insertion point, which increases the risk of anastomotic leakage. Circular stapling is advantageous for enhancing the margin quality and minimizing tension. However, its application proves to be more challenging in patients with obesity. The placement of the anvil is a primary challenge in circular stapling, and can be accomplished using either the Orvil technique or the reverse puncture technique. The Orvil technique, employing a "top-down" approach for releasing the anvil, streamlines the traditional anvil placement procedure. Nonetheless, the technique necessitates collaboration with a proficient anesthesiologist and poses a risk to the integrity of the esophageal mucosa. Additionally, the Orvil technique is contingent upon specific anatomical features of the patient’s pharynx and esophagus. In cases of gastrointestinal stenosis, the passage of the anvil could be obstructed, resulting in potential damage to the esophageal mucosa. The reverse puncture technique entails extracting the anvil through the anterior wall of the esophagus, and substituting the traditional purse-string suture with a linear cutting stapler, thereby streamlining the procedure. However, this approach entails inserting the suture needle into the abdominal cavity and the esophageal lumen, risking injury to the intra-abdominal tissues or esophageal mucosa. Furthermore, if the margins are found to be insufficient post-specimen retrieval, executing an additional esophageal resection via this technique proves difficult [9].

NAOKI HIKI et al. introduced a novel technique: The anvil's head was prepped with 2–0 sutures, and the anvil's tail was covered with a 10-cm length of nasogastric tube. Subsequently, the anvil sutures were secured to the nasogastric tube [10]. Nevertheless, this method is cumbersome, and there is a risk of intra-abdominal infection when passing a nasogastric tube through the esophagus into the abdominal cavity.

This study introduces a novel circular stapling technique that employs a catheter for guidance and positioning of the anvil. By circumventing oral and esophageal manipulation, this approach enhances the safety of the surgical procedure. As the esophagus remains intact during anvil insertion, the utilization of a linear cutting stapler for transection allows for the selection of an optimal incision line, preventing the residual esophagus from assuming a beak shape. Additionally, the elimination of purse-string suturing ensures a smooth texture in the tissues surrounding the lower section of the esophageal anvil. The traction afforded by the catheter facilitates the placement of the anvil, which is otherwise difficult to grasp. The sharp tip of the anvil poses a risk to the esophageal wall. Using the urinary catheter as a protective sheath for the anvil's tip minimizes the risk of damaging the esophagus and adjacent organs, thereby reducing the complexity of the procedure and the risk of esophageal leakage. By prioritizing the retrieval of the specimen followed by the extraction of the anvil from the esophagus, the sequence adeptly circumvents the necessity for intra-abdominal salvage surgery arising from insufficient margins. While the study did not report cases of inadequate margins during initial resections, the surgical protocol allows for further opening of the esophagus's right wall should the margins be insufficient. The urinary catheter can be employed to reposition the anvil toward the upper end of the esophagus, enabling supplementary esophageal resection.

The study encompassed 80 patients undergoing total gastrectomy, wherein the principal complications were pleural effusion and pneumonia. Pertaining to the gastrointestinal complications, which were the primary focus of this study, there were no instances of anastomotic leakage or bleeding among the patients. This implies that this stapling technique might offer advantages over linear stapling in terms of reducing anastomotic tension, which may in turn curtail the incidence of anastomotic leakage. Anastomotic stenosis emerged as the most prevalent complication in this study, representing 3.75% (3 cases), all of which were recorded among the initial 10 patients. This could be attributed to the excessive constriction of the bowel by utilizing the edges of gloves during the reconstruction, leading to the creation of smaller tissue incisions. As the proficiency of the surgeon increased, there was a significant reduction in the incidence of anastomotic stenosis.

This study possesses several limitations. It is a retrospective study, inherently accompanied by potential biases that cannot be entirely eliminated. Additionally, the small sample size renders it inconclusive for the broader population; larger-scale studies are essential for validating the safety and efficacy of this method. Moreover, the learning curve influenced the initial phase of the study, leading to an extended reconstruction time and the occurrence of anastomotic stenosis in three out of the first ten patients.

## Conclusion

This study proposes a catheter-guided anvil placement technique for circular stapler esophagojejunostomy in laparoscopic total gastrectomy, demonstrating the safety and feasibility of potential improvements. This method holds potential in mitigating complications like anastomotic leakage. However, rigorous prospective clinical studies involving a larger cohort are essential to ascertain its safety and efficacy.

## Data Availability

Data and materials of the current study are available.

## References

[1] Ferlay J, Soerjomataram I, Dikshit R, Eser S, Mathers C, Rebelo M, Parkin DM, Forman D, Bray F (2015) Cancer incidence and mortality worldwide: sources, methods and major patterns in GLOBOCAN 2012. Int J Cancer 136(5):E359-386 25220842 10.1002/ijc.29210

[2] Lin M, Huang CM, Zheng CH, Li P, Xie JW, Chen QY, Huang ZN (2017) Totally laparoscopic total gastrectomy for locally advanced middle-upper-third gastric cancer. J Vis Surg 3:46 29078609 10.21037/jovs.2017.03.17 PMC5638200

[3] Kodera Y, Yoshida K, Kumamaru H, Kakeji Y, Hiki N, Etoh T, Honda M, Miyata H, Yamashita Y, Seto Y et al (2019) Introducing laparoscopic total gastrectomy for gastric cancer in general practice: a retrospective cohort study based on a nationwide registry database in Japan. Gastric Cancer 22(1):202–213 29427039 10.1007/s10120-018-0795-0

[4] Washington MC, Mhalhal TR, Johnson-Rouse T, Berger J, Heath J, Seeley R, Sayegh AI (2016) Roux-en-Y gastric bypass augments the feeding responses evoked by gastrin-releasing peptides. J Surg Res 206(2):517–524 27884350 10.1016/j.jss.2016.08.057 PMC5125518

[5] Kawamura H, Ohno Y, Ichikawa N, Yoshida T, Homma S, Takahashi M, Taketomi A (2017) Anastomotic complications after laparoscopic total gastrectomy with esophagojejunostomy constructed by circular stapler (OrVil(™)) versus linear stapler (overlap method). Surg Endosc 31(12):5175–5182 28488177 10.1007/s00464-017-5584-z

[6] Robinson LA, Moulton AL, Fleming WH (1994) Techniques to simplify esophagogastric circular stapled anastomoses. J Surg Oncol 57(4):266–269 7990483 10.1002/jso.2930570411

[7] Aiolfi A, Sozzi A, Bonitta G, Lombardo F, Cavalli M, Campanelli G, Bonavina L, Bona D (2023) Short-term outcomes of different esophagojejunal anastomotic techniques during laparoscopic total gastrectomy: a network meta-analysis. Surg Endosc 37(8):5777–5790 37400689 10.1007/s00464-023-10231-6

[8] Lee S, Lee H, Song JH, Choi S, Cho M, Son T, Kim HI, Hyung WJ (2020) Intracorporeal esophagojejunostomy using a linear stapler in laparoscopic total gastrectomy: comparison with circular stapling technique. BMC Surg 20(1):100 32398072 10.1186/s12893-020-00746-3 PMC7218545

[9] Son SY, Cui LH, Shin HJ, Byun C, Hur H, Han SU, Cho YK (2017) Modified overlap method using knotless barbed sutures (MOBS) for intracorporeal esophagojejunostomy after totally laparoscopic gastrectomy. Surg Endosc 31(6):2697–2704 27699517 10.1007/s00464-016-5269-z

[10] Hiki N, Fukunaga T, Yamaguchi T, Nunobe S, Tokunaga M, Ohyama S, Seto Y, Muto T (2007) Laparoscopic esophagogastric circular stapled anastomosis: a modified technique to protect the esophagus. Gastric Cancer 10(3):181–186 17922097 10.1007/s10120-007-0433-8

